# Bathing adaptations in the homes of older adults (BATH-OUT): results of a feasibility randomised controlled trial (RCT)

**DOI:** 10.1186/s12889-018-6200-4

**Published:** 2018-11-26

**Authors:** Phillip J. Whitehead, Miriam R. Golding-Day, Stuart Belshaw, Tony Dawson, Marilyn James, Marion F. Walker

**Affiliations:** 1 Department of Social Work, Education and Community Wellbeing, Northumbria University, Room B018, Coach Lane Campus, Benton, Newcastle-Upon-Tyne, NE7 7XA UK; 2 Division of Rehabilitation and Ageing, University of Nottingham, The Medical School, Derby Road, Nottingham, NG7 2UH UK; 3 Adaptations and Renewals Agency, Nottingham City Council, Loxley House, Nottingham, NG2 3NG UK

**Keywords:** Housing, Adaptations, Older adults, Bathing, Prevention, Randomised controlled trial, Health economics

## Abstract

**Background:**

Housing adaptations have been identified as an important environmental and prevention intervention for older adults, which may improve health and quality of life. The onset of disability in bathing can act as a warning for further disability in other activities and may therefore be a judicious time-point for intervention. The aim of this study was to determine the feasibility of conducting a Randomised Controlled Trial (RCT) of bathing adaptations, to evaluate whether they improve older adults’ perceived health status and quality of life, prevent further functional deterioration, and reduce the use of other health and social care resources. This study was conducted in preparation for a powered RCT.

**Method:**

Eligibility criteria were aged > 65 and referred to local authority housing adaptations service for an accessible flush-floor shower. Participants were randomised to either usual adaptations (3–4 month wait) or immediate adaptations (no wait). Outcomes were assessed at 3, 6 and 9 months and included perceived physical and mental health status, health and social care related quality of life, independence in activities of daily living (ADL) and bathing, and falls. Data on costs and the use of health and social care resources were collected during follow-up in order to inform a definitive health economic evaluation.

**Results:**

Sixty participants were recruited and randomised, 31 to immediate adaptations and 29 to waiting list control. Mean age was 77(SD8), 58% women and 58% living alone. Follow-ups were completed with 90, 85 and 72% at 3, 6 and 9 months respectively. Adaptations were delivered to 65% of participants within the requisite timescales as there were delays with some privately owned properties. There were improvements from baseline in both groups on all outcome measures following the completion of the adaptations.

**Conclusions:**

This is the first RCT of housing adaptations in the UK. We demonstrated the feasibility of using a waiting list control, subject to minor alterations to the timescales for privately owned properties. A powered trial would evaluate the impact on older adults’ quality of life and investigate the impact of waiting times on functional outcomes and health and care resource use.

**Trial registration:**

ISRCTN14876332 Registered 12 July 2016.

**Electronic supplementary material:**

The online version of this article (10.1186/s12889-018-6200-4) contains supplementary material, which is available to authorized users.

## Background

People aged 65 and over spend more than 80% of their time at home [1] demonstrating the importance of the home environment to older adults’ health and wellbeing. Age related disabilities can lead to functional difficulties with everyday tasks and the home environment becoming burdensome. The World Health Organisation’s Active Ageing Policy Framework highlighted that barriers in the physical environment are of particular interest suggesting that ‘age friendly’ physical environments promote independence and may prevent further disease or disability [2]. Thus, housing and health are inextricably linked [3] and the state of the housing stock for older adults has important public health implications [4].

In a review of national and international evidence, housing adaptations were identified as one of the ‘ten most promising’ prevention interventions for older adults [5]. A housing adaptation is defined as “any permanent alteration carried out to a building with the aim of making it more suitable for a disabled person” [6]. The most common type of major housing adaptation for older adults is a bathing adaptation [7]. A ‘bathing adaptation’ usually involves the removal of the bath and replacement with an accessible, ‘level-access’ shower. Bathing adaptations may restore an older adult’s ability to bathe independently or enable a carer to support bathing. The onset of disability in bathing has been shown to be a particularly important event for older adults, often rapidly followed by disability in other daily activities [8], and even acting as a warning point for nursing home admissions [9]. Thus, it may represent a judicious time-point for intervention to prevent or delay further disability.

The Care Act 2014 has placed a duty on local authorities in England to provide services that prevent or delay the need for other health and social care services [10]. Although housing adaptations were identified as a promising prevention intervention for older adults [5] there are often lengthy waiting times of up to 2 years in some local authorities [11]. Care & Repair England have estimated that a delay of 1 year in providing a housing adaptation to an older person can increase homecare costs by £4000 [12]; this is comparable with the cost of providing a bathing adaptation. The need for a paid care worker to assist with personal care might be alleviated by the provision of an accessible showering facility enabling the person to manage independently.

Falls are the most common cause of injury related to deaths in people over the age of 75 in the UK, with up to 35% of people aged 65 and falling one or more times every year [13]. The estimated annual cost of falls to the NHS is over £2billion [14]. A significant protective factor for falls in older adults is environmental modification. Delays in the provision of housing adaptations may increase the risk of hospitalisation and associated costs [15]. Further research is needed into bathing adaptations specifically.

Research into the effects of housing adaptations has largely focussed on ‘minor’ adaptations (costing less than £1000) [16] such as grab rails, steps or threshold alterations and most studies have included a myriad of adaptations. A large RCT focussing on minor adaptations conducted in New Zealand reported a 26% reduction in the rate of injuries caused by falls in the intervention group [17]. Additionally, a longitudinal study conducted in Sweden found that for each month participants spent waiting for their housing adaptations their difficulty performing daily activities increased [18]. Despite these indications, there is no robust evidence of the effectiveness and cost effectiveness of major housing adaptations (such as accessible showers). A recent systematic review concluded that further research was needed, particularly using randomised or experimental designs [16]. A further systematic review of bathing interventions for older adults (not limited to adaptations) [19] identified only one study [20], and although the findings were promising, particularly in relation to reduced need for assistance from carers, the sample was small and the study was not randomised.

The aim of this study was to determine the feasibility of conducting a powered randomised controlled trial of bathing adaptations for older adults. However, there are ethical issues in randomising participants to a control group without the provision of adaptations; this study therefore used routine waiting times in order to form a control group. This study is in preparation for a definitive trial, with health economic evaluation, which will evaluate the effectiveness of bathing adaptations on health and quality of life outcomes. A definitive trial will also evaluate whether expedited adaptations (i.e. shorter waiting times) are associated with better outcomes and increased cost effectiveness.

## Methods

This paper has been written in accordance with the CONSORT 2010 statement: extension to randomised pilot and feasibility trials [21].

### Objectives

Specific objectives for this feasibility trial were: 1. To investigate whether the eligibility criteria were realistic; 2. To determine whether participants were willing to be randomised; 3. To calculate the attrition rate to inform a sample size calculation; 4. To determine whether the adaptations could be completed within the specified timescales; 5. To determine the suitability and participant completion of measures for use in a definitive trial; 6. To establish the feasibility and method of collecting information on costs of adaptations and health and social care resource use in order to inform a definitive health economic evaluation [22].

### Design

This was a single centre feasibility RCT. The RCT was a parallel group, two arm trial with a 1:1 allocation ratio intervention (immediate adaptations): waiting-list control.

### Setting

The study was conducted in one city council in England with a dedicated Adaptations and Renewals Agency (ARA). The agency coordinates and manages major adaptations (costing over £1000) for public sector (council owned) and private properties where a Disabled Facilities Grant (DFG) [23] is being used to fund or part-fund the adaptations. Between August 2016 and February 2017 staff at the ARA screened every referral to identify potential participants. ARA staff contacted potential participants by telephone to seek consent to pass their details onto the research team. Those who agreed were sent a mailed copy of the Participant Information Sheet and were then visited at home by a research assistant to provide a study explanation. Where participants were unable to consent due to mental capacity issues consultee opinion was sought as to whether they would have wished to take part if they did have capacity.

### Participants

Participants were adults, aged 65 or over, referred by a social care occupational therapy team member for provision of an accessible showering facility. Exclusion criteria were: referral for an accessible showering facility plus one or more other adaptations (e.g. hoist, ramp, lift), and priority ‘A’ referrals (those ‘fast-tracked’ based on clinical assessment).

Carers of participants were also approached for informed consent. We took a broad definition of ‘carer’ which was led by the participant and carer’s view of their role. This encompassed people who provided practical and/or emotional support, those who provided assistance with personal care and those who did not.

### Intervention and comparator

The intervention was the provision of an accessible showering facility: a flush floor anti-slip walk in ‘level access’ shower (which may also be termed a ‘wet room’). In circumstances where it was not possible to install a flush-floor (such as a high rise building) an easy access shower with a minimal access threshold was provided. The provision of the shower usually involved the removal of the existing bath, but could have been an alteration of a shower cubicle to make it more accessible. For publicly (council) owned properties the adaptation was paid for by the local authority; for private (owner occupier, privately rented and housing association owned) properties a means tested Disabled Facilities Grant (DFG) [23] was used to fund or part fund the adaptation in accordance with *The Housing Grants Construction and Regeneration Act 1996*. Participants in both groups received this intervention; however they were randomised to either:

- **Usual Adaptations Service (waiting-list control group)** Those randomised to the control group received the usual routine service provided by the Adaptations and Renewals Agency. This involved allocation to a project officer to begin processing the DFG application (if applicable) and planning the accessible showering facility after a 3 to 4 month wait.
- **Intervention (no waiting list)** Those randomised to the intervention group were allocated to a project officer to begin processing the DFG application (if applicable) and planning the accessible showering facility immediately. Thus, they did not go onto the routine waiting list.

### Outcomes

The main outcome for the study was to determine the feasibility of conducting a powered RCT. This was a composite of the feasibility objectives outlined above. We pre-specified our success criteria for proceeding to a main trial in the study protocol [22] as follows: 1. That the eligibility criteria were realistic to allow identification of 40 to 60 consenting participants; 2. That a minimum of 50% of eligible participants consented; 3. That a minimum of 70% of participants were followed-up at 6-months; 4. That a minimum of 70% of adaptations were completed within the follow-up timescales in both groups; 5. That a minimum of 80% of data completeness was achieved; 6. That sufficient data on costs and health and social care use could be collected in order to inform a definitive health economic evaluation.

A further objective was to determine the suitability of the measures used and whether they appeared responsive to change following the adaptations. Participant and carer outcomes were assessed at 3, 6 and 9 months post-randomisation. We altered the original study protocol to add an additional follow-up at 9 months, as we wanted to follow-up any participants who may not have had their shower installed by the 6 month visit. Participant outcomes were: perceived physical and mental wellbeing, health and social care related quality of life, personal activities of daily living, independence in bathing, perceived difficulty in bathing, perceived risk of falling, falls, number of care support hours, health and social care service usage. The outcome measures were: Short-Form 36 (physical and mental component scores) [24] EuroQol EQ5D-5 L [25], Adult Social Care Outcomes Toolkit^1^ (ASCOT) [26], Barthel Index [27] (bathing question analysed as a separate outcome), 0–100 scale for perceived difficulty in bathing, and the Falls-Efficacy Scale [28]. A purposely designed questionnaire gathered information on the use of other health and social care services, with particular emphasis on the use of homecare, residential care and time spent by paid or unpaid carers assisting with personal care.

Carer outcomes were: health related quality of life, perceived physical and mental wellbeing and caregiver strain. The outcomes measures were: EuroQol EQ5D-5 L [25], Short-Form 36 (physical and mental component summaries) [24], and Caregiver Strain Index [29]. We also gathered data on the carers’ use of health and social care services using a bespoke resource use proforma.

### Planned recruitment target and strategy

For a feasibility study, no formal sample size calculation is required. However, for this feasibility study the aim was to recruit between 40 and 60 participants (20 to 30 in each arm) to test the randomisation process and the feasibility of delivering the intervention in the proposed timescales. This sample size was decided upon as it is broadly consistent with the median sample size for UK feasibility trials which has been reported at 36 [30].

### Randomisation and blinding

Randomisation was generated online using a web-based randomisation programme www.sealedenvelope.com. Participants were individually randomised in random varying block sizes (sized in order to deliver the adaptations appropriately). Randomisation was stratified according to whether the property was publicly or privately owned. Randomisation was at a ratio of 1:1 (immediate adaptations to waiting list control). The allocation sequence was generated by www.sealedenvelope.com and members of the research team did not have access to it.

Baseline assessments were completed prior to randomisation. Follow-up assessment visits were completed by a research assistant (MGD) who was blind to allocation. Incidents of un-blinding were recorded and are reported below.

### Analysis

For feasibility outcomes, descriptive statistics were calculated based on analysis of the trial screening and recruitment log, follow-up rates, and routine adaptations data gathered by the Adaptations and Renewals Agency. Participant and carer outcome data was analysed by intention to treat, and participants were analysed according to their group assignment irrespective of whether they received the adaptation. Summary statistics, estimates of effect and confidence intervals were used. Effectiveness testing was not carried out as this is not appropriate for feasibility work [21].

## Results

### Recruitment and participant flow

**Fig. 1:**
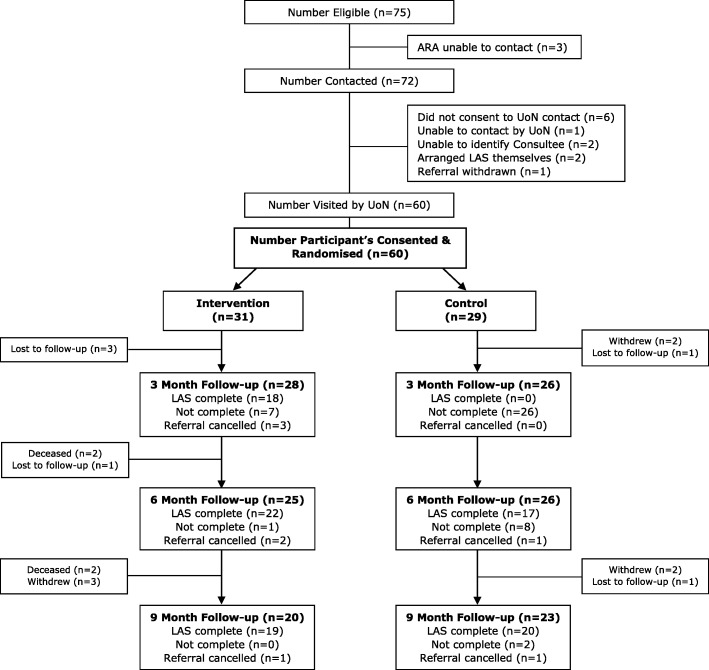
Participant Flowchart

The trial opened for recruitment on 15th August 2016 and closed on 2nd March 2017. The final follow-up visit for the study was completed on 29th November 2017. Fig. 1 shows the recruitment figures and the flow of participants through the study. In total, 75 participants met the eligibility criteria and the Adaptations and Renewals Agency made contact with 72 of them. Sixty-six (92%) agreed for their details to be passed to the research team and 100% of those who received the study information agreed to participate. Sixty participants were randomised, 31 to immediate adaptations and 29 to waiting list control. Fig. 1 also shows attrition: 54 (90%) were followed-up at 3 months, 51 (85%) at 6 months, and 43 (72%) at 9 months and were included in the analysis.

Forty participants had a carer and 23 (58%) were recruited. Seventy percent of recruited carers lived with the participant compared with 50% overall. Carers were more likely to be recruited into the study when they were present on the initial research visit, some participants did not consent for the research team to make contact with the carers when they lived elsewhere stating that they did not want to overburden the carer. Eighteen carers were followed-up at 3 months (78%), 16 (70%) at 6 months and 13 (57%) at 9 months.

### Baseline data

The demographic characteristics and medical details of participants and carers are shown in Table 1. The groups were well matched on participant demographic characteristics, although there was a slight preponderance of women in the waiting list control group, and people with neurological conditions as the primary diagnosis in the immediate adaptations group. In the immediate adaptations group there were more carers who were White British and retired and they were, on average, 14 years older. Table 1 also shows the time that participants had waited, prior to randomisation, from their referral to occupational therapy for the assessment. On average, participants had waited just under 4 months prior to recruitment and this was slightly longer in the waiting list control group.

**Table 1:** Participant and Carer Demographic Characteristics and Medical Details

	Participant Immediate Adaptations (*n* = 31)	Participant Waiting List Control (*n* = 29)	Carer Immediate Adaptations (*n* = 12)	Carer Waiting List Control (*n* = 11)
Age Mean (SD)	77.74 *(7.64)*	76.34 *(7.65)*	72.67 *(10.66)*	58.64 *(15.04)*
Gender				
Male	15 *(48%)*	10 *(34%)*	3 *(25%)*	4 *(36%)*
Female	16 *(52%)*	19 *(66%)*	9 *(75%)*	7 *(64%)*
Ethnicity				
White British	25 *(81%)*	25 *(86%)*	10 *(83%)*	6 *(55%)*
Other	6 *(19%)*	4 *(14%)*	2 *(17%)*	5 *(45%)*
Employment				
Retired	31 *(100%)*	29 *(100%)*	10 *(83%)*	6 *(55%)*
Employed	0	0	2 *(17%)*	2 *(18%)*
Unemployed	0	0	0	3 *(27%)*
Property Tenure				
Council	17 *(55%)*	16 *(55%)*		
Owner Occupied	9 *(29%)*	9 *(31%)*		
Housing Association	5 *(16%)*	4 *(14%)*		
Living Arrangement				
Alone	19 *(61%)*	16 *(55%)*		
With other(s)	12 *(39%)*	13 *(45%)*		
Carer				
Within household	11 *(36%)*	9 *(32%)*		
External to household	10 *(32%)*	10 *(34%)*		
No carer	10 *(32%)*	10 *(34%)*		
Carer Assists with				
Domestic ADL only	9 *(43%)*	9 *(47%)*		
Both Personal and Domestic ADL	12 *(57%)*	10 *(53%)*		
Primary Diagnosis				
Musculo-skeletal	16 *(52%)*	19 *(66%)*		
Neurological	9 *(29%)*	3 *(10%)*		
Respiratory	3 *(10%)*	3 *(10%)*		
Frailty	2 *(6%)*	4 *(14%)*		
Mental Health	1 *(3%)*	0 *(0%)*		
Consultee Opinion				
Yes	3 *(10%)*	4 *(14%)*		
No	28 *(90%)*	25 *(86%)*		
Time from OT referral to randomisation (days) Mean (SD)	103.71 *(92.08)*	125.07 *(105.64)*		
Time from ARA referral to randomisation (days) Mean (SD)	14.70 *(5.94)*	15.21 *(6.53)*		

Baseline measures are shown in Table 2 (participants) and Table 3 (carers). Participant groups were well matched on baseline measures although those in the immediate adaptations group scored slightly higher on all measures and there were slightly more participants in the waiting list control group bathing independently at baseline. Carers in the control group had slightly better scores for perceived health and quality of life at baseline.

**Table 2:** Participant Baseline and Outcome Measures

		Bl	3 M	6 M	9 M
Short-Form 36 Physical Component Summary Mean (SD)	Immediate Adaptations	29.03 *(7.41)*	30.09 *(8.21)*	32.29 *(9.57)*	32.76 *(11.83)*
	Waiting List Control	27.46 *(6.75)*	25.02 *(6.54)*	27.17 *(7.78)*	30.65 *(7.76)*
Short-Form 36 Mental Component Summary Mean (SD)	Immediate Adaptations	46.02 *(9.82)*	49.47 *(8.98)*	50.47 *(7.97)*	53.34 *(8.18)*
	Waiting List Control	44.65 *(9.42)*	44.55 *(10.75)*	47.52 *(10.03)*	48.64 *(10.10)*
EQ5D-5 L Mean (SD)	Immediate Adaptations	0.44 *(0.25)*	0.53 *(0.25)*	0.63 *(0.24)*	0.59 *(0.30)*
	Waiting List Control	0.41 *(0.28)*	0.39 *(0.28)*	0.52 *(0.28)*	0.58 *(0.28)*
EQ5D Perceived Health Mean (SD)	Immediate Adaptations	45.81 *(19.92)*	50 *(26.84)*	61.16 *(22.95)*	63.45 *(24.21)*
	Waiting List Control	41.55 *(17.88)*	42.11 *(19.45)*	51.73 *(22.76)*	60.22 *(23.86)*
ASCOT Mean (SD)	Immediate Adaptations	0.74 *(0.18)*	0.78 *(0.18)*	0.82 *(0.16)*	0.85 *(0.16)*
	Waiting List Control	0.71 *(0.21)*	0.70 *(0.23)*	0.74 *(0.22)*	0.82 *(0.19)*
Barthel Index Mean (SD)	Immediate Adaptations	15.68 *(3.40)*	15.82 *(4.67)*	15.28 *(5.30)*	15.7 *(4.91)*
	Waiting List Control	15.64 *(4.96)*	15.00 *(5.45)*	15.68 *(5.12)*	16.73 *(4.40)*
Perceived Ease of Bathing Mean (SD)	Immediate Adaptations	27.58 *(21.90)*	67.14 *(35.36)*	80.6 *(24.34)*	89 *(18.04)*
	Waiting List Control	26.03 *(21.93)*	19.81 *(21.19)*	59.81 *(39.99)*	83.26 *(30.02)*
Bathing Independent	Immediate Adaptations	9 *(29%)*	19 *(68%)*	17 *(68%)*	14 *(70%)*
	Waiting List Control	13 *(45%)*	10 *(38%)*	17 *(65%)*	18 *(78%)*
Short Falls Efficacy Scale^a^ Mean (SD)	Immediate Adaptations	20 *(4.97)*	18.63 *(5.48)*	17.48 *(5.68)*	16.95 *(5.92)*
	Waiting List Control	20.14 *(4.64)*	20.92 *(4.21)*	18.84 *(4.99)*	17.36 *(5.00)*

^**a**^Higher score indicates poorer outcome

**Table 3:** Carer Baseline and Outcome Measures

		Bl	3 M	6 M	9 M
Short-Form 36 Physical Component Summary Mean (SD)	Immediate Adaptations	42.85 *(9.39)*	44.78 *(7.83)*	46.62 *(6.87)*	39.29 *(11.95)*
	Waiting List Control	44.98 *(11.20)*	40.81 *(9.39)*	43.47 *(11.11)*	41.51 *(11.85)*
Short-Form 36 Mental Component Summary Mean (SD)	Immediate Adaptations	43.84 *(10.24)*	44.28 *(8.20)*	44.69 *(11.16)*	44.95 *(7.08)*
	Waiting List Control	47.14 *(11.08)*	48.03 *(10.68)*	50.05 *(9.81)*	50.86 *(13.44)*
EQ5D-5 L Mean (SD)	Immediate Adaptations	0.74 *(0.18)*	0.80 *(0.09)*	0.81 *(0.15)*	0.77 *(0.13)*
	Waiting List Control	0.77 *(0.23)*	0.77 *(0.13)*	0.81 *(0.18)*	0.74 *(0.25)*
EQ5D Perceived Health Mean (SD)	Immediate Adaptations	61.25 *(18.23)*	66.11 *(22.61)*	64.29 *(21.49)*	55 *(14.14)*
	Waiting List Control	61.36 *(22.70)*	64.44 *(25.06)*	68.89 *(22.19)*	65.63 *(22.27)*
Caregiver Strain Index^a^ Mean (SD)	Immediate Adaptations	7.17 *(2.08)*	6.89 *(2.15)*	6.86 *(2.19)*	7.4 *(2.51)*
	Waiting List Control	7.55 *(2.77)*	6.67 *(2.83)*	7 *(2.87)*	6.63 *(2.67)*

^a^Higher score indicates poorer outcome

### Feasibility outcomes

#### Eligibility, recruitment and attrition

It was possible to identify a pool of potentially eligible participants, between 11 and 12 per month. Eighty-three percent of those who were eligible consented to take part and were randomised. Our recruitment target was 40 to 60 participants in 8 months; we recruited 60 participants in just over 6 months. Overall attrition was low and was balanced between the groups. Eighty-five percent were followed-up at 6 months. The main reason for attrition was loss to follow-up as we were unable to make contact with six participants over the 9 month period due to moving or changing their contact details. Five participants withdrew between the 6 and 9 month follow-ups, the nine-month follow-up was added as a study amendment and participants were asked for further consent for this additional visit and five chose not to continue with the study. Overall, we vastly exceeded our pre-specified targets for identifying eligible participants, recruitment and attrition rates.

#### Provision of intervention within specified timescales

The mean (SD) times to completion are shown in Table 4. Privately owned properties took longer than those that were publicly owned, due to the time spent processing the DFG applications. Housing associations took the longest time from randomisation to completion in both immediate and waiting list control groups. Fig. 1 also shows the participants who had their adaptations completed in the requisite timescales. At 3 months 64% of adaptations were complete in the immediate adaptations group. There were delays with two owner-occupied properties, all five housing association properties, and three participants cancelled the referral due to their assessed financial contributions following the means test. By 6 months 88% were complete in the immediate adaptations group. At 6 months 65% of adaptations were complete in the waiting list control group. There were delays with four privately owned properties, all four housing association properties, one publicly owned property, and one referral was cancelled due to the assessed financial contribution. In total, eight of the 27 privately owned properties did not proceed with the adaptations due to their assessed financial contribution; some of these were lost to follow-up and did not continue with the study.

**Table 4:** Time from Randomisation to Adaptation Completion (days)

	Immediate Adaptations	Waiting List Control
Public – Council Mean (SD)	48.35 *(14.57)*	154.57 *(17.76)*
Private – Owner Occupier Mean (SD)	114.5 *(42.38)*	204.38 *(47.94)*
Private – Housing Association Mean (SD)	131.4 *(33.48)*	286.75 *(45.19)*

#### Suitability and completion of outcome measures

Table 3 shows the participant outcomes at baseline, 3, 6 and 9 months. All participant outcome measures improved from baseline at 3 months in the immediate adaptations group, compared to minimal changes or slight worsening in the control group. At 6 months, these improvements from baseline were largely mirrored in the control group across all measures following the installation of their adaptations. At 9 months both groups had improved from baseline, with the immediate adaptations group overall slightly higher than the control group as they were at baseline. Overall there was a 98% completion of the measures which ranged from 93 to 100% for individual measures.

Table 5 shows the data on falls at baseline and follow-up; this data was collected retrospectively from the participants at each of the three time-points. There was a reduction in falls in both groups from baseline, this applies to the number of participants reporting one or more falls, the median falls per participant, and the total number of falls per group. Fewer participants reported falls in the immediate adaptations group during the follow-up period (*n* = 13) compared to the waiting list control group (*n* = 15) and there were fewer falls overall in the immediate adaptations (*n* = 33) compared to the waiting list control (*n* = 65). Although there was one outlier in the control group who reported falling every day (90 times at baseline, 3 months and 6 months) there were fewer falls in the immediate adaptations group both including and excluding this data.

**Table 5:** Participant Falls

Immediate Adaptations					Waiting List Control			
	n	Participants reporting one or more fall	Median (IQR) Falls	Total Falls	n	Participants Reporting one or more fall	Median (IQR)	Total Falls
Baseline	31	18	2 (1–3)	46	29	16	2 (1–3)	41^a^
3 M	28	8	1 (1–1.5)	11	26	9	2 (1–10)	31^a^
6 M	25	7	1 (1–2)	10	26	12	1.5 (1–3)	19^a^
9 M	20	6	1.5 (1–2)	12	23	5	1 (1–2)	15

^a^One participant reported falling every day during follow-up (*n* = 90). We removed this participant as an outlier from the total falls figures. The participant was not followed-up at 9 months

With regard to carers, Table 3 shows the outcomes at baseline 3, 6 and 9 months. There were improvements in perceived health status (mental and physical) and quality of life following the completion of the adaptations in both groups. There was a reduction in carer strain following baseline.

Table 6 shows the change from baseline for participants in the immediate group compared to the control group at 3 months, including the 95% confidence intervals. The direction of change on all outcomes was in favour of the immediate adaptations group, although the confidence intervals are wide reflecting the small sample size. Thus, it appears that the measures are sensitive to change in the population and suitable for the bathing adaptations intervention.

**Table 6:** Change from Baseline 3 Months (Citizen)

	Immediate Adaptations -Waiting List Control Difference (SE) 95% CI
PCS	3.39 (1.48) 0.41, 6.36
MCS	3.41 (2.08) -0 .77, 7.60
EQ5D-5 L	0.09 (0.05) -0.003, 0.18
EQ5D Perceived Health	4.56 (6.03) -7.54, 16.66
ASCOT	0.06 (0.04) -0.02, 0.14
Barthel Index	0.43 (0.56) -0.69, 1.56
Perceived Ease of Bathing	43.75 (7.29) 29.12, 58.38
Short Falls Efficacy Scale	−2.34 (0.84) -4.03, −0.64

#### Health economic and cost data collection

The average cost of the adaptations was £4878.46 across all participants, however, it was £4625.77 in the immediate adaptations group compared to £5131.15 in the control group a difference of just over £500. It is possible that overall costs were rising over time such that the contractors increased their prices for the work that was completed later (the waiting list control group). It is also possible that the additional works were added to the referrals in the waiting list control group during the wait, possibly due to deterioration. However, it could also be a chance finding due to the small sample size. All three issues may have been factors and warrant further investigation.

We were able to collect the requisite data on use of health and social care services during the follow-up period which would inform a definitive health economic analysis alongside the EQ5D-5 L data. We demonstrated that the measures used and their collection methods would be suitable for roll out into a definitive trial. We collected a range of data on contacts with health and social care services including in-patient and out-patient visits, community and home appointments, this data for baseline and 3 months follow-up is included in Additional file 1: Tables S1 and S2. Although participants had some difficulties recalling this information, they were on the whole able to provide it. We particularly focused on information related to assistance with personal care and time spent by paid or unpaid carers (Additional file 1: Tables S3 and S4); there was a particular reduction in the need for assistance from unpaid carers aiding with bathing following the installation of the shower.

#### Study processes: Blinding of the outcome assessments

The research assistant collecting outcome data was asked to report instances of un-blinding. They reported 15 definite instances at 3 months, ten in the immediate adaptations and five in the control group. They were also asked to make their ‘best guess’ and correctly guessed the allocation for 46/51 (90%) participants at 3 months. However, the CONSORT extension [21] states that incidents of un-blinding are likely to be due to the effectiveness of the intervention rather than the success of the blinding. We have reported this finding as it was specified in our protocol [22] which preceded the CONSORT extension.

## Discussion

We found that it was feasible to conduct an RCT of bathing adaptations for older adults in collaboration with a local authority housing adaptations partner. We demonstrated a high level of participant engagement evidenced by rapid recruitment and low levels of attrition. It was possible to collect a complete set of outcome data across a comprehensive range of outcomes and collect the cost and resource use data which would be needed for a definitive economic evaluation. All outcome measures appeared responsive to change as improvements in all outcome measures were observed in both groups following the completion of the bathing adaptations; these indicative trends warrant further investigation in a powered RCT. However, there were difficulties in delivering the adaptations within the 3 month timescales for some privately owned properties and all housing association properties, this would necessitate changes to the timescales in a definitive trial and is discussed further.

The principal strength of this study is that, to our knowledge, it is the first to use a randomised method to evaluate any type of housing adaptation in the UK and the first to evaluate major adaptations worldwide. The use of the routine waiting list as a control facilitated a comparison between those that had and did not have bathing adaptations; it would not be not be possible to randomise people to not have bathing adaptations for ethical reasons. This is also one of the first studies to focus specifically on the effects of an intervention to improve or maintain independence in bathing. Although there is an indication that bathing disability may be a precursor to further disability for older adults [8], there is a dearth of research investigating the effects of the preventive impact of bathing interventions [19]; this study is an important first step in this regard which may have important public health and preventative implications. The principal limitation is that the study was conducted in a single site and thus we have not established feasibility at other sites. However, we have identified key factors for success in terms of recruitment and intervention delivery which should be feasible in other sites, subject to careful site selection for a multi-centre trial.

Although there is a dearth of evidence for housing adaptations using randomised and experimental designs [16], our indicative findings are consistent with the wider literature which suggests the effects on quality of life are positive and important to older adults [6, 15, 16]. As part of the BATH-OUT study we also conducted a concurrent qualitative interview study to explore the lived experience of bathing adaptations for older adults. The initial findings support the selection of outcome measures used in this study as older adults emphasised the impact of the adaptations on: their physical functioning within the home; overall confidence and quality of life; feeling clean; choice and control; feeling of safety (Whitehead PJ, Golding-Day M: The lived experience of bathing adptations and the BATH-OUT study for older adults and their carers: A qualitative interview study, in preparation). Consultations with our Public Involvement Group, all of whom have lived experiences of bathing adaptations, indicated that physical functioning and impact on physical health status were of primary importance; they believed that physical improvements led onto improved quality of life as a secondary outcome. We therefore propose to use the Physical Component Summary of the SF-36 as the primary outcome in the powered trial.

Compared to other feasibility trials our findings on eligibility, recruitment, attrition, and the suitability of outcome measures are strong and are promising for a future, powered study. Recruitment rates were higher than expected and were in excess of the usual for clinical trials and rehabilitation studies at 83% of those eligible. The positive recruitment rate may be due to the 50% chance of expedited adaptations and the possibility of not going onto the routine waiting list; overall, participants expressed a preference for allocation to immediate adaptations. It is also likely that the lack of an ‘experimental’ treatment increased the acceptability of the trial; participants in both groups were due to receive the adaptation, therefore there was limited uncertainty as to what would happen to them if they entered the study. It is also possible that the high level of engagement within the local research team, which included the manager of the adaptations service, was a factor in the recruitment success. In the qualitative interviews participants reported that the face-to-face follow-up visits with the research assistant also encouraged study participation.

Based on our findings we suggest that a powered trial is feasible. However, our target was to complete 70% of adaptations within the allocated timescales [22] and we achieved 65%. There were particular difficulties with completing the adaptations within the timescales in housing association properties due to the additional administrative procedures in administering the grant. We suggest that a powered trial should be conducted in housing adaptations services with slightly longer waiting times for the control period with outcomes collected at a later time-point. At 4 months the majority of adaptations would have been completed in the immediate adaptations group, facilitating the comparison between the groups. Additionally, 30% of privately owned properties did not proceed with the adaptations following the assessed means test due to their financial contribution; we also suggest that the informal means test should be completed for private properties prior to randomisation to reduce loss to follow-up and lack of fidelity for participants not proceeding with the adaptations. Furthermore, the main trial should incorporate an internal pilot with stop/go criteria in order to determine that the adaptations are being completed within the requisite timescales.

## Conclusion

Although housing adaptations have been identified as one of the ten ‘most promising’ prevention services for older adults [5] there is a paucity of high quality evidence of their effect on older adults’ health and quality of life and the impact on their use of health and social care services. This study has demonstrated that a powered RCT using a novel waiting list control group is feasible, subject to minor changes in trial design. Given the importance of the home environment for older adults’ health and wellbeing, and the significance of the onset of bathing disability in the life course, we suggest that the results of a powered RCT in this area would have important public health and policy implications. A definitive trial has the potential to determine important preventative outcomes of bathing adaptations for older adults and evaluate whether increased waiting times are associated with lower functional outcomes, health inequalities and a reduction in cost effectiveness.

### Trial status

Data collection and analysis are complete, this is a results paper. The trial is registered ISRCTN14876332.

## Additional file

Additional file 1: Health Economic Data Tables. Participant’s use of health and social care resources at baseline and 3 month follow-up. (DOCX 22 kb)

## Abbreviations

ADL: Activities of Daily Living
ARA: Adaptations and Renewals Agency
CONSORT: Consolidated Standards of Reporting Trials
DFG: Disabled Facilities Grant
RCT: Randomised Controlled Trial

## Footnotes

1 The ASCOT is designed to capture the effects of social care interventions and includes domains such as personal cleanliness, safety, choice and control and dignity. It also incorporates questions about the feelings associated with having assistance with particular activities, which is different to health-related quality of life.
