# Supplementary material for: Bathing adaptations in the homes of older adults (BATH-OUT): results of a feasibility randomised controlled trial (RCT)
Source: BMC Public Health. 2018 Nov 26;18:1293. doi: 10.1186/s12889-018-6200-4 (PMC6257950; doi:10.1186/s12889-018-6200-4)
Supplement: Supplementary file 1 — Health Economic Data Tables. Participant’s use of health and social care resources at baseline and 3 month follow-up. (DOCX 22 kb) [file 12889_2018_6200_MOESM1_ESM.docx]

**Health Economic Table S1: Participant Baseline Use of Health and Social Care Services**

|  | **Intervention**  **(n= 31)**  *Mean (Std Dev)* | | **Waiting List Control**  **(n=29)**  *Mean (Std Dev)* | |
| --- | --- | --- | --- | --- |
| **Visit GP Surgery** | n =8 | 3.9 *(1.96)* | n =12 | 9.9 *(16.6)* |
| **Visit GP Home** | n = 4 | 2 *(0.82)* | n = 6 | 2.2 *(2.04)* |
| **Visit GP Phone** |  |  | n = 1 | 1 |
| **Visit Practice Nurse** | n = 6 | 1.5 *(0.84)* | n = 10 | 1.6 *(0.84)* |
| **Visit Community Nurse** | n = 8 | 3.9 *(1.96)* | n = 12 | 9.9 *(16.6)* |
| **Visit OT Local Clinic** |  |  |  |  |
| **Visit OT Home** | n = 30 | 1 *(0.18)* | n = 29 | 1.1 *(0.37)* |
| **Visit Physio Local Clinic** |  |  |  |  |
| **Visit Physio Home** | n = 3 | 2 *(1.7)* | n = 3 | 1 *(0)* |
| **Visit Community Social Services** | n = 1 | 1 |  |  |
| **Visit Other NHS Services** | n = 1 | 2 | n = 1 | 1 |
| **Hospital Outpatient (any reason)** | n = 17 | 2.5 *(1.46)* | n = 15 | 1.3 *(0.62)* |
| **Hospital Physio** | n = 1 | 8 | n = 1 | 2 |
| **Hospital OT** | n = 1 | 1 |  |  |
| **Hospital A&E** | n = 6 | 1.2 *(0.41)* | n = 1 | 1 |
| **Hospital Other** |  |  |  |  |
| **Inpatient Length of stay (days)** | n = 13 | 12.9 *(14.58)* | n = 4 | 9.25 *(7.93)* |
| **Community Hospital** |  |  |  |  |
| **Intermediate Care Bed** |  |  |  |  |
| **Nursing Home NHS** |  |  |  |  |
| **Nursing Home Private** |  |  |  |  |
| **Residential Care Home** | n = 2 | 52 *(53.74)* |  |  |
| **Stay with Family/Friends** | n = 1 | 21 | n= 1 | 6 |
| **Other** |  |  |  |  |

**Health Economic Table S2: Participant 3 Month Use of Health and Social Care Services**

|  | **Intervention**  **(n=31)**  *Mean (Std Dev)* | | **Waiting List Control**  **(n=29)**  *Mean (Std Dev)* | |
| --- | --- | --- | --- | --- |
| **Visit GP Surgery** | n = 11 | 1.5 *(0.69)* | n = 16 | 1.6 *(0.81)* |
| **Visit GP Home** | n = 5 | 1.2 *(0.45)* | n = 2 | 2 *(1.4)* |
| **Visit GP Phone** | n = 1 | 1 | n = 1 | 1 |
| **Visit Practice Nurse** | n = 2 | 1 *(0)* | n = 9 | 2.6 *(3.61)* |
| **Visit Community Nurse** | n = 7 | 4.1 *(2.91)* | n = 5 | 4 *(4.58)* |
| **Visit OT Local Clinic** |  |  |  |  |
| **Visit OT Home** | n = 10 | 1 *(0)* | n =3 | 1 *(0)* |
| **Visit Physio Local Clinic** | n = 1 | 12 |  |  |
| **Visit Physio Home** | n = 1 | 4 | n = 3 | 6 *(6.93)* |
| **Visit Community Social Services** | n = 1 | 3 |  |  |
| **Visit Other NHS Services** | n = 4 | 3.5 *(4.36)* | n = 1 | 12 |
| **Hospital Outpatient (any reason)** | n = 13 | 1.9 *(1.44)* | n = 14 | 2 *(2.42)* |
| **Hospital Physio** |  |  |  |  |
| **Hospital OT** |  |  |  |  |
| **Hospital A&E** | n = 4 | 1 *(0)* | n = 1 | 1 |
| **Hospital Other** |  |  |  |  |
| **Inpatient Length of stay (days)** | n = 6 | 8.3 *(8.48)* | n = 1 | 21 |
| **Community Hospital** |  |  |  |  |
| **Intermediate Care Bed** |  |  |  |  |
| **Nursing Home NHS** |  |  |  |  |
| **Nursing Home Private** |  |  |  |  |
| **Residential Care Home** |  |  | n = 1 | 4 |
| **Stay with Family/Friends** |  |  | n = 1 | 6 |
| **Other** |  |  | n = 1 | 12 |

**Health Economic Table S3: Participant Baseline Assistance from Paid and Unpaid Carers**

|  | **Intervention**  **(n= 31)**  *Mean (Std Dev)* | | **Waiting List Control**  **(n= 29)**  *Mean (Std Dev)* | |
| --- | --- | --- | --- | --- |
| **Paid Care Worker** | | | | |
| No 0  Yes 1 | 23  8 | | 22  7 | |
| **How Often (Daily)** | n = 6 | 2.2 *(1.17)* | n = 6 | 2.2 *(1.17)* |
| **How Often (Weekly)** | n = 2 | 1.5 *(0.71)* | n = 1 | 2 |
| **How Often (Monthly)** |  |  |  |  |
| **How much time per visit (minutes)** | n = 8 | 84.4 *(92.1)* | n = 7 | 49.3 *(33.22)* |
| **Carer assists with Personal Care?**  No 0  Yes 1 | 24  7 | | 22  7 | |
| **How much time (minutes)** | n = 7 | 31.4 *(16)* | n = 7 | 26.4 *(6.27)* |
| **Carer (Family/Friend)** | | | | |
| No 0  Yes 1 | 4  27 | | 3  26 | |
| **How Often**  Daily 0  Weekly 1  Other 2 | 20  5  2 | | 18  6  2 | |
| **How much time per visit (minutes)** | n = 26 | 392.7 *(371.87)* | n = 26 | 410.19 *(491.29)* |
| **Carer assists with Personal Care?**  No 0  Yes 1 | 18  13 | | 21  8 | |
| **How much time (minutes)** | n = 13 | 40.77  *(25.89)* | n = 8 | 46.3 *(26.29)* |
| **Anyone taken time off work?**  No 0  Yes 1 | 29  2 | | 28  1 | |
| **Number of Days** | n = 1 | 1 | n = 1 | 12 |
| **Carer also provides care for someone else?**  No 0  Yes 1 | 27  4 | | 26  3 | |

**Health Economic Table S4: Participant 3 Month Assistance from Paid and Unpaid Carers**

|  | **Intervention**  **(n=28 )**  *Mean (Std Dev)* | | **Waiting List Control**  **(n= 26 )**  *Mean (Std Dev)* | |
| --- | --- | --- | --- | --- |
| **Paid Care Worker** | | | | |
| No 0  Yes 1 | 20  8 | | 19  7 | |
| **How Often (Daily)** | n = 5 | 2.4 *(1.14)* | n = 6 | 2.3 *(1.21)* |
| **How Often (Weekly)** | n = 3 | 2.7 *(2.08)* | n = 1 | 2 |
| **How Often (Monthly)** |  |  |  |  |
| **How much time per visit (minutes)** | n = 8 | 253.13 *(482.5)* | n = 7 | 83.6 *(122.43)* |
| **How much time per visit (minutes)**  ***Outlier removed**** | n = 7 | 83.57  *(57.35)* | n = 7 | 83.6  *(122.43)* |
| **Carer assists with Personal Care?**  No 0  Yes 1 | 22  6 | | 19  7 | |
| **How much time (minutes)** | n = 6 | 31.7 *(14.72)* | n = 7 | 25 *(6.45)* |
| **Carer (Family/Friend)** | | | | |
| No 0  Yes 1 | 4  24 | | 3  23 | |
| **How Often**  Daily 0  Weekly 1  Other 2 | 17  5  2 | | 15  7  1 | |
| **How much time per visit (minutes)** | n = 23 | 388.7 *(324.56)* | n = 23 | 345 *(404.34)* |
| **Carer assists with Personal Care?**  No 0  Yes 1 | 24  4 | | 18  8 | |
| **How much time (minutes)** | n = 4 | 33.8  *(7.5)* | n = 8 | 38.1 *(25.35)* |
| **Anyone taken time off work?**  No 0  Yes 1 | 28  0 | | 25  1 | |
| **Number of Days** |  |  | n = 1 | 3 |
| **Carer also provides care for someone else?**  No 0  Yes 1 | 26  2 | | 24  2 | |
| *1 participant had increased to 24 hour carer and was removed as an outlier | | | | |
